# Exploring the Mental Model of Cattle Farmers in Disease Prevention and Control Practices

**DOI:** 10.3390/vetsci7010027

**Published:** 2020-02-28

**Authors:** Yong Suit-B, Latiffah Hassan, Steven Eric Krauss, Siti Zubaidah Ramanoon, Peck Toung Ooi, Abd Rahaman Yasmin, Jonathan Epstein

**Affiliations:** 1 Department of Veterinary Laboratory Diagnostics, Faculty of Veterinary Medicine, Universiti Putra Malaysia, Serdang 43400, Malaysia; gem_cysb@hotmail.com (Y.S.-B.); latiffah@upm.edu.my (L.H.); noryasmin@upm.edu.my (A.R.Y.); 2 Department of Professional Development and Continuing Education, Faculty of Educational Studies, Universiti Putra Malaysia, Serdang 43400, Malaysia; 3 Department of Medicine and Surgery of Farm & Exotic Animal, Faculty of Veterinary Medicine, Universiti Putra Malaysia, Serdang 43400, Malaysia; sramanoon@upm.edu.my; 4 Department of Veterinary Clinical Studies, Faculty of Veterinary Medicine, Universiti Putra Malaysia, Serdang 43400, Malaysia; ooi@upm.edu.my; 5 EcoHealth Alliance, New York, NY 10001, USA; epstein@ecohealthalliance.org

**Keywords:** social epidemiology, qualitative, cattle farmers, mental model, disease prevention

## Abstract

Farmers play an integral role in minimizing disease threats and managing ongoing diseases on their farms. Various environmental factors influence the decision-making processes of farmers. Deciphering the mental models of farmers allows us to understand the motivations and reasons behind disease prevention and control choices. This study aimed to explore the mental models of cattle farmers in implementing disease prevention and control practices. Using qualitative in-depth, semi-structured interviews, seven cattle farmers from a university’s foster farm extension program were sampled. Interview transcripts were analyzed using inductive content analysis. Results revealed 23 dimensions comprising the mental model of cattle farmers. The dimensions were conceptualized under four major themes. Farmers were most influenced by perceived risk of disease, perceived effectiveness and benefits of disease prevention and control practices, experience, knowledge and emotions, subjective norms and perceived economic loss. The decision-making processes of farmers are complex and are influenced by various factors. While additional research is needed to confirm the findings using quantitative methods and larger sample sizes, insights gained from the study can be used as inputs to tailor communication and training strategies for improved disease prevention and control interventions.

## 1. Introduction

There is an estimated 710,481 cattle in Malaysia [1]. Sixty percent (60%) are owned by smallholders and medium traditional farmers [2] with an average of 21 cattle per farm [3]. Cattle are raised in different production systems, varying from intensive, semi-intensive, tree-crop integration and extensive systems [4,5]. Semi-intensively or extensively raised cattle are free to roam, enhancing the probability of contact with infectious materials between diseased herds, cattle and wildlife or other domestic animals. The ongoing strategy to improve food security by transforming small-scale ruminant farms to viable commercial-scale farms [6] increases farm density and animal movements. This agriculture intensification may enhance disease risks to humans and animals, especially when accompanied by poor management and hygiene [7].

Outbreaks of zoonotic diseases such as brucellosis and tuberculosis in cattle have been reported locally [8,9]. The test-and-slaughter eradication policy implemented in 1979 for bovine brucellosis led to a targeted reduction of brucellosis prevalence in the country until 1998, when the prevalence began to rise again due to multiple risk factors [8]. Contact with wildlife [10], unauthorised movement or importation of infected animals and lack of farmers’ cooperation in disease control are challenges in preventing brucellosis [11]. The convenience of unnecessary documentation for importing cattle and attraction of cheaper herds prompts certain farmers to import herds from disease-endemic areas or countries illegally [11,12]. Other zoonotic diseases reported in cattle are leptospirosis, meliodiosis, Q fever, rabies, trypanosomosis, bartonellosis and fascioliasis [13,14,15,16,17,18,19].

Farmers play an integral role in implementing disease prevention and control practices. Unfortunately, farmers are not always compliant with best practices or recommended disease control programs [20]. Despite considerable awareness of herd health programs, local dairy farmers in Malaysia have low compliance, especially in disease monitoring and biosecurity [21]. A study of local dairy farmers using the fuzzy index model found that the practices for herd health and biosecurity management were only moderate (0.447) when compared to recommended practices with high variation among farmers (0.14–0.91). A large gap exists among farmers in adopting ideal disease prevention and control practices, influenced by socioeconomic factors such as insufficient capital, limited knowledge and access to information and technology, high operational cost, short staff turnover and disinterested staffs, difficulty obtaining market information and limited marketing channel and inadequate support from the local veterinary services [22].

Social epidemiology refers to the holistic approach of integrating herd health management and understanding of farmers’ behaviours in preventive medicine [23]. The integration of social sciences helps public health researchers unravel the reasons behind farmers’ decision-making and behaviour [24]. A proposed model on the adoption of biosecurity efforts in farmers suggests that socio-contextual factors and psychosocial concepts such as threat perceptions, cognitions, attitudes and beliefs, disease specificity, social norms, perceived costs, efficacy, motivation, framing and resilience influence biosecurity engagements [25]. Past research has shown that financial gains and trusted veterinarians were positive drivers for implementing zoonotic control programs in cattle farmers, whereas discouraging social norms, low self-efficacy, knowledge barriers and cultural and economic pressures were negative drivers [26].

Examining the decision-making processes of farmers through a mental model approach provides greater insight into how farmers decide and act the way they do toward disease prevention [25,27]. A mental model is an internal representation of the mind created to interpret the environment [28]. Values, knowledge, experiences and emotions influence farmers’ mental models, which directly impact decision-making processes and actions [29,30]. Insights into farmers’ mental models can improve communication strategies, policy-making and advisory interventions by reinforcing positive practices, addressing key knowledge gaps and reinforcing the credibility of communications and their sources [24,27].

Against this backdrop, the present study aims to: (1) explore the mental model of Malaysian cattle farmers in implementing disease prevention and control practices and (2) identify other challenges to broaden our understanding on the circumstances surrounding disease prevention and control.

## 2. Materials and Methods

Interviews were conducted by the primary author, who is a veterinarian and researcher. The author is familiar with cattle farming and disease prevention and control practices. This study was carried out using a qualitative descriptive approach based on the naturalistic paradigm [31]. All subjects gave their informed consent for inclusion before they participated in the study. The study was conducted in accordance with the Declaration of Helsinki, and the protocol was approved by the Ethics Committee of University Putra Malaysia (#JKEUPM-2019-127) on April 16, 2019. In-depth, semi-structured interviews were conducted between April and June 2019. Purposive sampling was used to recruit cattle farm managers from a list of extension foster farms, i.e., ”Ladang Angkat” in Selangor and Negeri Sembilan that were attached to the Faculty of Veterinary Medicine, UPM. These partner farms received regular visits and veterinary services from clinicians from the University Veterinary Hospital, UPM, and, in turn, provide clinical training opportunities for veterinary students [32]. Participants voluntarily enrolled, and consent was obtained. Seven out of the eight cattle farmers approached via phone call agreed to participate in the data collection. An honorarium of MYR30 (USD7) was given as a token of appreciation following each interview. Participants had no previous relationship with the interviewer. Participants were informed that the interviewer was a veterinary Master’s degree candidate. Face-to-face interviews were conducted mostly in English and Malay and were audio-recorded and then transcribed. Each interview lasted 15–60 min.

An interview guide (Supplementary Guide 1) was designed by the research team, which included researchers with backgrounds in sociology, epidemiology and veterinary medicine. The guide contained open-ended questions and probes to explore subjective meanings and motivations related to farmers’ disease prevention and control practices. No formal pre-test of the interview guide was performed. Consistent with a sequential, qualitative research data analysis approach, interview questions were refined, and additional probing questions added following analysis of each subsequent interview [33]. Participants were encouraged to elaborate and share their experiences; the interviewer sought clarification and posed follow-up questions on topics related to the study objectives. The interviewer began the interviews by asking general questions to build rapport. Questions posed aimed at gathering information about the interviewee and his respective farm, such as years of farming experience, herd size and husbandry practices. This was followed by subsequent questions pertaining to knowledge and experience of zoonotic diseases, impact of disease outbreak, general disease prevention and control practices, decision-making factors that influenced disease prevention, treatment or control strategies, challenges and needs in preventing diseases, general challenges and needs of the industry and any related issues. Probes for decision-making factors comprised financial means, risk of disease, food safety, drugs, accuracy of test, friends’ opinions, law requirement, love for animals and animal welfare. The final part of the interview gathered demographic data on each farmer’s age and education level. Field notes related to observations of farmers’ behaviours or reflective information were taken.

**Figure 1:**
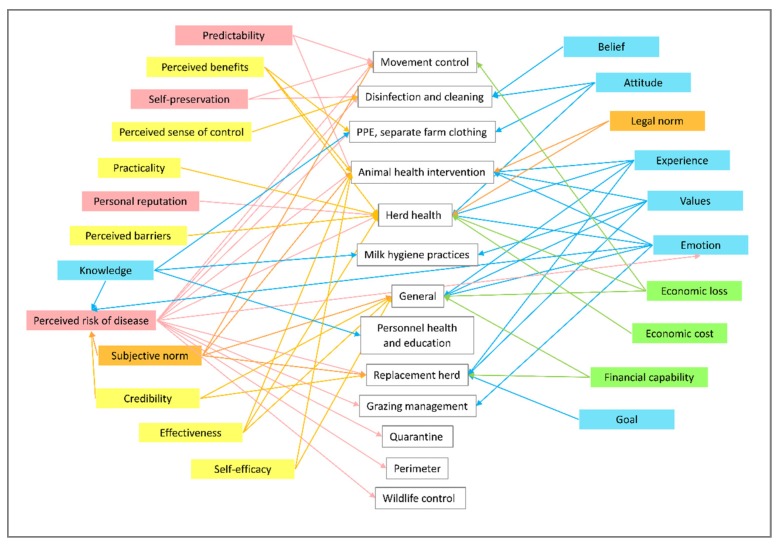
Conceptual map of cattle farmers’ mental model, demonstrating relationships between dimensions and disease prevention and control practices. PPE: personal protective equipment.

Inductive content analysis was performed to code, categorize and abstract themes from the transcripts. This form of analysis was most suitable to explore farmers’ insights on disease prevention and control practices and because limited knowledge on decision making of these farmers is known [34]. Transcripts were read and re-read for full appreciation of the interview contents. Some audio recordings were re-played to better grasp farmers’ replies [35]. Sentences and text passages that corresponded to study objectives were highlighted and coded. Open coding was done by first identifying and labeling disease prevention and control practices, followed by motivations or reasons for performing or not performing those practices. Transcripts were re-read, reviewed and coded through an iterative process. Codes and original corresponding text passages were transferred to a coding sheet and categorized into groups according to similarities. A conceptual map was created linking each disease prevention and control practice to the codes of motivation or reasons for those practices (Figure 1). The mapping revealed the thought processes behind the adoption or non-adoption of those practices. Direction of arrows showed how one dimension of the mental model affects other dimensions or practices. Further abstraction of categories was carried out in order to organize the categories into more conceptual themes to create a simplified mental model map [34,36].

## 3. Results

### 3.1. Farmers’ Characteristics

A total of seven farmers participated in the study. All farmers were male (7/7) in the 40–69 age group. Most (4/7) had a tertiary education and above. All farmers were managing open housing systems at the time of the study. Two farmers managed dairy and beef cattle production, three farmers managed dairy cattle production and two farmers managed beef cattle production. Average herd size was 118 cattle, ranging from 40–200. Some (5/7) farmers reared other livestock, such as buffalo, goat and horse. Most (4/7) farmers had more than 30 years of experience in farming. Tuberculosis and brucellosis were common zoonotic diseases farmers were familiar with. Some farmers (4/7) had experienced tuberculosis or brucellosis on their farms (Table 1).

### 3.2. Mental Model

Six categories of disease prevention and control practices with 21 practices were identified from the interviews (Table 2).

**Figure 2:**
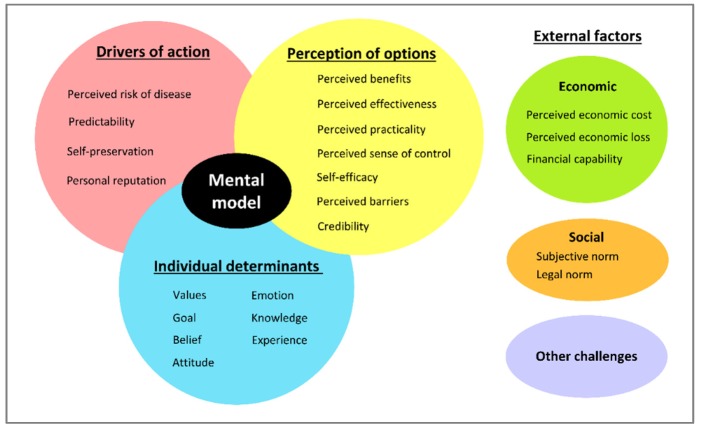
Simplified conceptual map of cattle farmers in implementing disease prevention and control practices.

Through content analysis, 23 categories or dimensions of the mental model were determined. A conceptual map representing the farmers’ mental model was constructed to demonstrate relationships between dimensions and specific practices (Figure 1). A simplified conceptual map with four themes was created (Figure 2).

#### 3.2.1. Drivers of Action

Drivers of action are motivations or reasons that act as triggers to implement a particular practice. Drivers of action identified through analysis included perceived risk of disease, predictability, self-preservation and preserving one’s personal reputation. The most discussed factor was perceived risk of disease, especially in preventing outbreaks through purchase or selection of new herds, as expressed by all farmers (7/7). Perceived risk of disease was evaluated in terms of susceptibility and severity. Farmers highlighted the importance of purchasing new herds from credible sources, obtaining health clearance from the veterinary authority, disease screening, quarantine or selecting replacements from their own herd to minimize the risk of introducing diseases to their farm.

Must be careful. When you buy new cows and bulls, you have to do blood test first. Some people have Brucella, they won’t tell you. Then the sick animals come to your farm, breed with our cows, they will get Brucella.

(F2)

You see, first is you cannot buy diseased animals. If the animal is carrying disease, the disease is there. Get good animals, clean animals, from abroad, not local. You cannot source animals locally now.

(F5)

A few farmers (2/7) demonstrated the need to prevent introducing diseased animals and spreading of disease from infected animals to healthy animals from nearby farms during outbreak situations. They would cease purchase of new animals, cull infected animals or perform drastic measures like cull and restock the entire herd.

We cannot bring animals from outside. We just keep whatever we have and we test them again. We must cull the infected ones. Other than that, we can’t do much. Or, we can cull the entire herd.

(F1)

Some farmers (4/7) were driven by perceived risk of disease to disinfect and clean. Some of these actions were supported through the understanding of disease transmission routes. Farmers expressed concern about fomite transmission, such as on clothing, boots and vehicles.

Every day we make sure that we throw all feces away to make sure the floor is clean and wash their legs and nails because diseases come from their nails.

(F4)

When outbreaks are happening, we will definitely put some sacks of lime and all at the main entrance.

(F5)

Preventing contact with free-roaming cattle and wildlife was important for one farmer due to the presence of these animals around his farm. This triggered the farmer to create perimeter drains, contact the veterinary authority for wildlife control and prevent his cattle from grazing in the fields. The farmer also expressed fear of this particular disease risk.

Wild cows can jump a 4-foot drain. I make big perimeter drains and STILL they come in! And then there’s a lot of wild boars. Wild boars carry a lot of diseases, blood parasite diseases. So it’s very challenging.

(F3)

No grazing, I cut and carry. Too scared to let them out to graze. When you know this group of friends has all kinds of sickness, you won’t want to mix with them. You know that all the village cows are there, why would you want to let your cows graze?

(F3)

#### 3.2.2. Perception of Practice Options

Farmers’ perceptions of practice options are evaluations on disease prevention and control choices. Analysis revealed several categories, including perceived benefits, perceived effectiveness, credibility, self-efficacy, perceived sense of control, practicality and perceived barriers. Most of the categories were given equal coverage by the farmers during the interviews as motivations for certain practices. Herd health management decisions for a few of the farmers (2/7) were influenced by perceived effectiveness, benefits, barriers and practicality. Some considerations were the effectiveness of vaccinations, perceived impracticality or lack of facilities to perform vaccinations and clean water for better quality milk and living.

I think the effectiveness of vaccines and drugs are important. But the vaccines for FMD are not practical. The hassle of catching every cattle for vaccination every six months is too much work. You need 5 people to catch because there’s no proper cattle crush.

(F7)

The tube well is very expensive. But it’s okay, I’m only thinking of improving the quality of the milk, the quality of the cows, their life, you know, give them the best.

(F3)

Extra supplementation or drug usage was influenced by self-efficacy, perceived effectiveness and benefits. This action was supported by the belief that vitamin supplementation is beneficial to enhance immunity.

During an outbreak, we will spend on medicine and vitamins. We will increase vitamin supplementation to increase their antibody based on their body weight. We are confident to give this because we have a weighing scale.

(F4)

#### 3.2.3. Individual Determinants

Experience, knowledge, values, goals, beliefs, attitudes and emotions were individual determinants affecting practice decisions. Experience, knowledge and emotions were most common in influencing practices. Farmers’ experiences prompted both general disease control practices and practices related to replacements of herds, herd health and animal health interventions. The farmers were careful to avoid reliving negative past experiences. When asked why he chose to cull and restock his entire herd, one of the farmers responded, “Because we don’t know—I had tuberculosis in my farm last time. It’s very contagious.” (F3).

A few of the farmers (2/7) changed their purchasing strategy to source from local suppliers or selected a replacement herd from their own herd due to the unfortunate experience of introducing diseased animals into their herd. One farmer expressed loss of trust in relying on a government-subsidized scheme for the procurement of new animals as a consequence of a negative experience.

We don’t buy from neighboring countries. We only buy from local reputable suppliers with proper records. Because animals that we bought last time died. We don’t know why. It looks physically healthy. When we bought, it did not have any records of drugs given and health.

(F4)

We better grow ourselves, buy animals ourselves without assistance. My farm had tuberculosis from the pregnant heifers introduced under the previous animal subsidized scheme. Foot-and-mouth disease broke out in the first week, and later many had tuberculosis.

(F5)

Knowledge of diseases was important to encourage farmers (4/7) to practice good milk hygiene practices, conduct disinfection, separate farm shoes, carry out personnel health checks and educate personnel on zoonotic diseases. Different types of knowledge illustrated included how good milk hygiene affects bacteria loads in milk, disease transmission through close contact and fomites and the severity of zoonotic diseases like brucellosis.

If you go to any farm, you must be clean, you must have medicine, and most important is to spray disinfectant. If this farm has disease, the germs sometimes touch your things, your boots, and your shoes. So when you go to their farm, the germs will spread. FMD can spread very fast. You can see the signs in 12 h–10 h.

(F2)

Workers must go for medical check-ups. Sometimes, foreigners have tuberculosis. Sometimes, the cows can also be infected. When we spit, maybe the cow comes in contact.

(F2)

I’ve learned about (brucellosis) from my time studying at university but I’ve never seen a case. But it’s a serious disease, so it’s important that people are aware and that my workers are aware.

(F7)

Emotions of fear or worry elicit certain herd and animal health interventions, as well as grazing management and other general practices. One farmer (F1) put it simply as, “If we don’t spend now, we will regret it later.” Another replied, “Scary—must be careful. When an outbreak is happening, you have to check the cows and bulls you take in. Blood test first.” (F2)

One worried farmer was willing to cull all his animals and restock the entire herd in order to have peace of mind.

If I have my old cows here, and I add in new cows, all these cows will also get sick! I am also very worried. When you get a headache, you take Panadol, isn’t it? Headache gone. That’s what I’m doing now. I’m making myself feel comfortable that I’m bringing in new cows, all disease free.

(F3)

#### 3.2.4. External (Social and Economic)

External social factors that guide farmers’ disease prevention and control practices were subjective and legal normative behaviors (norms). Subjective norms were the most apparent. This dimension affected movement control, animal health intervention, replacement of herds and general practices. Many farmers (4/7) had good relationships with veterinarians and would actively seek for their advice or follow their recommendations.

Usually our animal department will have workshops or programs with vets and lecturers from UPM. The measures you must take to prevent, etc. I mainly get my advice from UPM doctors. I can call them anytime and they can advise me to do this, do that.

(F3)

We also had to slaughter some that were not having diseases because it was recommended by the veterinary authority, just dispose.

(F5)

A few of the farmers (2/7) mentioned that news of disease outbreaks from other farmers or social media platforms were influential to increasing vigilance.

Through word of mouth, my friends will tell me careful of some cows, there’s disease.

(F3)

We join a lot of breeders Facebook groups. From there we get information when they update on current disease status.

(F4)

The external economic factors discussed by the farmers in determining practice options were financial capability, perceived economic loss and perceived economic cost. Perceived economic loss from the act of culling animals, reduced production, reduced profit margin and absence of compensation were most noticeable in farmers (4/7). These stimulated decisions on general practices and herd health, such as increased monitoring efforts and movement control, such as prohibiting outsiders onto farms.

When we have diseases, we have to put animals to sleep. It will affect my milk production and income. I have to source for milk outside and profit margin will be less. If we don’t spend to prevent diseases now, we will regret later. The loss will be bigger, very big!

(F1)

So after I buy the new cows, I will be more careful. I won’t even let students come inside. Because you all visit a lot of farms also, you can carry the disease, you see. And you know, the cows are very expensive, RM 8000, 9000, 10,000. Loss of livestock is a big deal for me. We have no insurance. If anything happens, we cannot get compensation.

(F3)

However, one farmer said that he did not feel particularly affected by disease outbreaks on other farms, as he was already experiencing monthly losses. This farmer expressed that his primary motivation for farming was not economic profit but the intrinsic value of owning a beautiful cattle farm.

This is not really a business [for me]. I lose money every month so nothing matters to me. But if the stock is wiped out then I’ll be very upset.

(F7)

I lived abroad for a few years and I like animal farming. I think the cattle farms are very beautiful. So I wanted something like that.

(F7)

#### 3.2.5. External (Other Challenges)

The external challenges that emerged from the interview data affecting disease prevention and control include expensive vaccinations, lack of vaccinations for some diseases, difficulty in keeping farms clean at all times, maintaining foot dip when it rains, disease threats from unmanageable free-roaming cattle and wild boars, perceived lack of support from the veterinary authority, limited ability for screening tools to detect disease at an early stage in apparently healthy new animals, lack of laboratories to confirm diagnoses, importation of unhealthy animals by other farmers, absent or minimal compensation for culled animals, insufficient disease outbreak information and humid climates. Other industrial challenges include unprofitable business, expensive land, lack of grazing land and unreliable or insufficient employees.

## 4. Discussion

This study aimed to gain insight into the underlying reasons for cattle farmers’ implementation practices related to disease prevention and control. This process of unraveling the rational assessments pertaining to farmers’ decisions is important to better understand behaviors that experts may see as irrational. Hence, there is a need to bridge the gap between farmers, veterinarians and agricultural extension educators. It is recognized that farmers’ motivations to continue or change are beyond the simplistic notion of “economic rationality” and are, rather, governed by complex sets of core values which can be discerned through social science studies [37].

Our analysis revealed that the farmers’ perceived risk of disease greatly affected their decisions to prevent and control actual disease outbreaks. This finding mirrors those of other studies [38,39]. All seven farmers in our study were highly concerned about introducing diseases to their farms via the purchase of new herds. As a result, they took extensive precautionary actions. Past studies show that the purchase of animals from established dealers was ranked as the highest risk factor for introducing disease by dairy farmers [39]. In the current study, only one farmer raised the issue of perceived risk of disease from wildlife. Although this may be influenced by individual observations or experience, it may indicate other farmers’ lack of knowledge on the subject. An emphasis on mitigating the risk of disease transmission between wildlife and livestock is crucial, as 70% of emerging zoonotic diseases originate from wildlife [40].

Perceived risk of disease also interacted with important individual determinants of the mental model, such as experience, knowledge and emotions. Limited knowledge on modes of transmission and perception about the risk of zoonotic diseases are factors that widen the awareness-practice gap [41]. This resonated with farmers’ thought processes of being aware of potential diseases and the modes of transmission. Studies conducted on farmers in other settings have revealed that experiences, values and knowledge influence mental models of farming and, subsequently, learning, problem solving and decision-making [29]. In the current study, negative past experiences with disease outbreaks enriched the farmers’ knowledge on diseases and triggered negative emotions. Emotions are an undeniable force that impact decision-making [42]. Emotions of fear and worry were positive drivers in enhancing certain practices in order to have peace of mind, such as a farmer who took drastic action to cull his entire herd, including even animals that tested negative for disease prevalence.

The current study further found that farmers were more likely to enforce certain practices that they deemed to be more effective and beneficial. Educating farmers in order to improve comprehension of complex information will lead to better management decisions [43]. This was supported by a farmer who stated that he would more willingly invest in better facilities or practices when the benefits are clear. Nonetheless, despite understanding the importance of certain practices, some farmers were inhibited by perceived practicality or structural limitations.

Many farmers expressed that veterinary advice from the University and the Department of Veterinary Services was vital in guiding disease prevention and control practices. The high reliance on veterinary advice may stem from the long-established relationship and trust between farmers and veterinarians and the veterinary authority. Frequent interactions between farmers and veterinarians are highly recommended and often lead to the achievement of extension goals. The role of veterinarians goes beyond disseminating technical information; they need to be skillful communicators and proactive in order to build strong relationships [44]. Consequently, farmers will be stimulated and empowered to make informed decisions [45].

Perceived economic loss was a prominent factor in motivating farmers to prevent and control diseases. However, economic motivations may not always apply to all farmers because of various behavioral types. A study found five distinct patterns of behavioral motivations for farming. Farmers in the categories of “family orientation” (29.6%) and “business/entrepreneur” (25.9%) were more inclined to prioritize economic factors in order to pass on a viable business to the next generation and avoid debt, respectively. The opposite applies to “life-stylers” (21.5%), “enthusiasts/hobbyists”, (16.6%) and “independent/small farmers” (6.4%), who are less concerned with the financial aspects of farming [46]. This explains why one farmer was unaffected economically by a disease outbreak, as he is an “enthusiast/hobbyist” who does not depend on farming for income but farms for enjoyment and satisfaction.

Consistent with the preponderance of qualitative research literature that centers study goals around the generation of new hypotheses resulting from in-depth textual analysis rather than to generalize findings to a large population, the current study’s findings cannot be generalized to all cattle farmers in Malaysia [47]. The current study’s small sample size was limited to farmers from the Veterinary Faculty’s foster farm program, which only consists of small to medium-intensive and semi-intensive farms. No farmers from integrated and extensive production system farms were interviewed. Variability in farm size, production system, farming environment and challenges, experience of diseases, institutional support and cultures could also affect the mental models of farmers. Moreover, farmers in this study benefit from regular visits and good relationships with veterinarians from the university; thus, the farmers are presumably well-informed. Nevertheless, in-depth insights generated from this study can be used to formulate testable hypotheses for larger future studies. Further studies aimed at comparing the mental models of farmers attached with university extension services and those without attachments can help identify gaps to improve education and extension services to farmers.

## 5. Conclusions

The decision-making processes of farmers are complex and influenced by numerous elements. Perceived risk of disease was a prominent factor to motivate farmers to prevent and control disease. Preventing diseases by the introduction of new herds was a priority among all participating farmers. Good veterinarian-farmer relationships were imperative to enhance the receptivity of farmers to advice and be empowered to make informed decisions. Knowledge acquired from various sources broadened and deepened the understanding of disease risk and perception of practice options. This often led farmers to the behavioral intention to prevent and control diseases, but poor perception of practicality or structural limitations inhibited such actions. Economic factors influenced certain practices but may not be applicable to all farmers due to distinctive motivations for farming. Recognizing the unique mental models of specific farmers or specific types of farmers will be advantageous for veterinarians and agricultural extension educators to tailor effective messages and elevate persuasiveness for improved disease preventions and control interventions. Challenges beyond farmers’ control also need to be addressed to support farmers’ disease preventions and control efforts. Further research is needed with larger and more diverse samples to confirm the findings.

## Figures and Tables

**Table 1:** Characteristics farm and cattle farmers interviewed.

Production Type	Production System	Cattle Herd Size	Other Farmed Animals	Years of Farming Experience	Familiar Zoonotic Disease	Experienced Tb or Bru Outbreak
Dairy	Semi-intensive	80	Buffalo, goats	>30	Tb, Bru	Yes
Dairy	Intensive	60	-	>20	Tb	Yes
Dairy	Intensive	150	Buffalo	>40	Tb, Bru	Yes
Beef	Semi-intensive	200	-	>40	Tb, Bru	Yes
Beef	Intensive	40	Horse, goats	>10	Tb, Bru	No
Beef & Dairy	Semi-intensive	200	Buffalo	>30	Tb, Bru	No
Beef & Dairy	Intensive	100	Buffalo	>10	Bru	No

Tb: tuberculosis and Bru: brucellosis.

**Table 2:** Disease prevention and control practices identified from the interviews.

No.	Practices	Category of Practices
1	Disinfection and cleaning	Biosecurity
2	Personal protective equipment (PPE)/separate farm clothing	
3	Movement control	
4	Replacement herd	
5	Quarantine	
6	Perimeter	
7	Wildlife control	
8	Grazing management	
9	Clean water	Herd health
10	Disease screening	
11	Animal care and monitoring	
12	Isolation	
13	Vaccination	
14	Veterinary	Animal health intervention
15	Supplementation	
16	Drug use	
17	Culling	
18	Milk hygiene practices	Milk hygiene practices
19	Personnel education	Personnel health and education
20	Personnel health check	
21	General disease control practices	General

## Supplementary Materials

The following are available online at https://www.mdpi.com/2306-7381/7/1/27/s1, Guideline 1: Interview Guide for In-Depth Interviews with Cattle Farmers.
