# Supplementary material for: Exploring the Mental Model of Cattle Farmers in Disease Prevention and Control Practices
Source: Vet Sci. 2020 Feb 28;7(1):27. doi: 10.3390/vetsci7010027 (PMC7158665; doi:10.3390/vetsci7010027)
Supplement: Supplementary file 1 [file vetsci-07-00027-s001.pdf]

Supplementary Material

Exploring the Mental Model of Cattle Farmers in Disease Prevention and Control Practices

Yong Suit-B[1], Latiffah Hassan[1], Steven Eric Krauss[2,*], Siti Zubaidah Ramanoon[3], Peck Toung, Ooi[4], Abd Rahaman Yasmin[1], Jonathan Epstein[5]

1 Department of Veterinary Laboratory Diagnostics, Faculty of Veterinary Medicine, Universiti Putra Malaysia, 43400, Serdang, Malaysia;
2 Department of Professional Development and Continuing Education, Faculty of Educational Studies, Universiti Putra Malaysia, 43400, Serdang, Malaysia;
3 Department of Medicine and Surgery of Farm & Exotic Animal, Faculty of Veterinary Medicine, Universiti Putra Malaysia, 43400, Serdang, Malaysia;
4 Department of Veterinary Clinical Studies, Faculty of Veterinary Medicine, Universiti Putra Malaysia, 43400, Serdang, Malaysia;
5 EcoHealth Alliance, New York, 10001, United States

* Correspondence: lateef@upm.edu.my; Tel.: +603-89468243/+603-97698243

Guide S1: Interview Guide for In-Depth Interviews with Cattle Farmers

Ice-breaking questions

1. How many years have you reared cattle?
2. How many livestock are there in your current farm?
3. What is the purpose of the livestock?

Questions

1. Have you heard of zoonotic disease @ diseases that can be transmitted between animals and humans? Can you tell me what do you know about zoonotic diseases?
2. Have you had any seminar or courses on zoonoses?
   Besides seminar/courses, where else do you get information from (eg, internet, friends, etc)?

3. What are the zoonotic disease outbreaks that you are familiar with or have experienced?
   If yes, do any of your friends have the same experience?
   If no, have your friends experienced any diseases transmitted from animals?
   (reflect on common zoonotic disease outbreaks in the past)
4. How did the outbreak affect you?
   (probe: emotionally, business/financially, husbandry decisions, farm management, farm biosecurity, herd health, personal hygiene, personal protective equipment)
5. Can you tell me about what do you do to prevent and control diseases in your farm?
   Has it changed over the years? (if yes – what are some of the changes, if not, probe why not)
   *even though the risk is high, why don't you make any changes?
   *how will you make sure that [_______outbreak] won't happen again?
6. When you want to do anything to prevent or control zoonotic disease, what will you consider? How will you decide? What will you think about when deciding?
   (probe: financial means, high/low risk of disease, food safety, effectiveness of drugs, accuracy of tests, opinion from friends, law requirement, love for animals, animal welfare, others?)
7. What are the challenges or limitations and needs during and after a disease outbreak?
8. Is there anything else that you would like to add to the topic that we've discussed today?

Demographic

1. What is your age? ______________

2. Sex __________ *M / F*

3. What is your education level? (optional) Illiterate, Literate, Primary, Secondary, University
